# Supplementary material for: MicroRNA Profiles in Monocyte-Derived Macrophages Generated by Interleukin-27 and Human Serum: Identification of a Novel HIV-Inhibiting and Autophagy-Inducing MicroRNA
Source: Int J Mol Sci. 2021 Jan 28;22(3):1290. doi: 10.3390/ijms22031290 (PMC7865382; doi:10.3390/ijms22031290)

Supplementary Information

MicroRNA Profiles in Monocyte-derived Macrophages Generated by Interleukin-27 and Human Serum: Identification of a Novel HIV-inhibiting and Autophagy-inducing MicroRNA

Tomozumi Imamichi, Suranjana Goswami, Xiaojun Hu,
Jun Yang, Sylvain Laverdure, Ju Qiu, Qian Chen,
Brad T. Sherman, and Weizhong Chang

S. Figure S1.

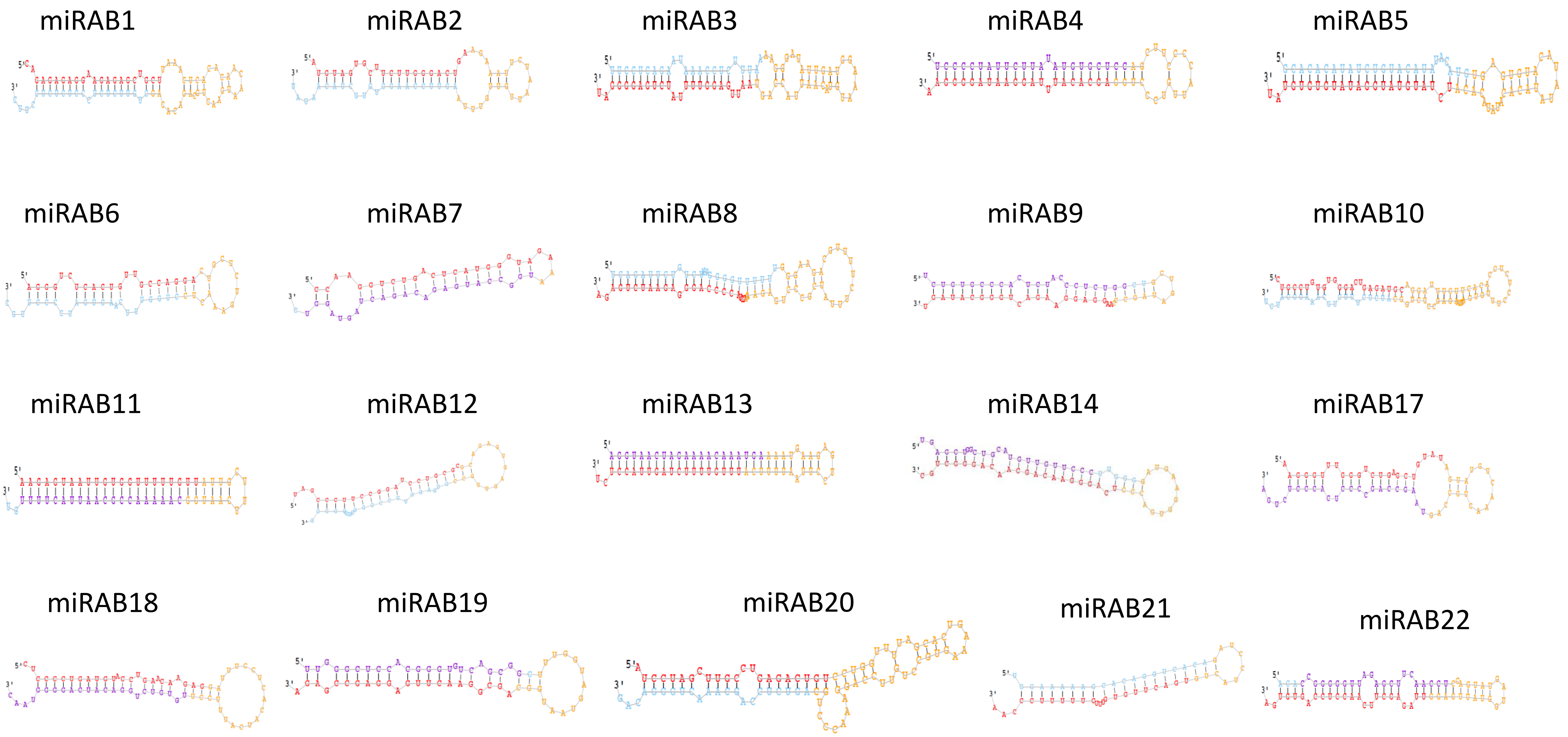
miRAB1
miRAB2
miRAB3
miRAB4
miRAB5
miRAB6
miRAB7
miRAB8
miRAB9
miRAB10
miRAB11
miRAB12
miRAB13
miRAB14
miRAB17
miRAB18
miRAB19
miRAB20
miRAB21
miRAB22

S. Figure S1. (continued)

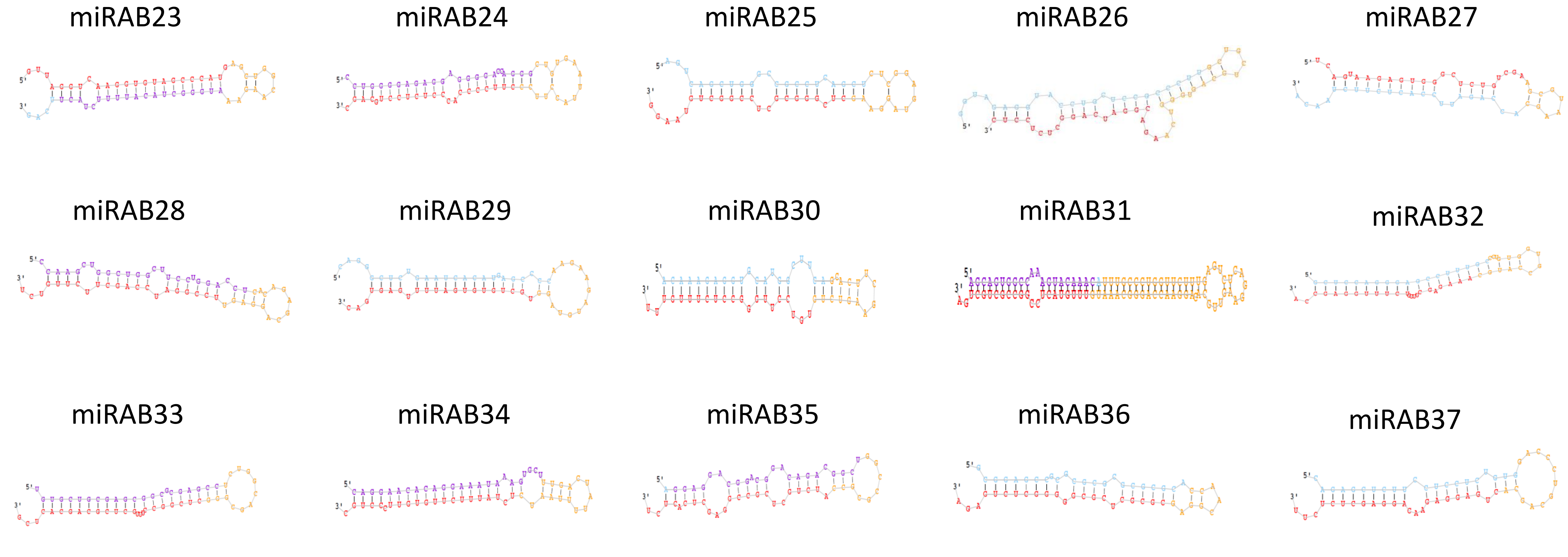

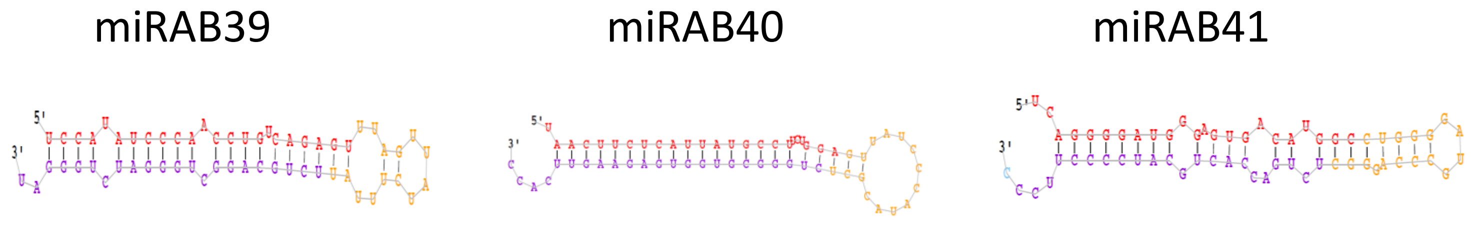

S. Fig 1. Secondary structures of the novel miRNAs. All novel miRNAs were able to form stem-loop hairpin structures. Red indicates the mature sequence; blue indicates the star sequence; yellow indicates loop structure, purple indicates star sequence.

S. Figure S2

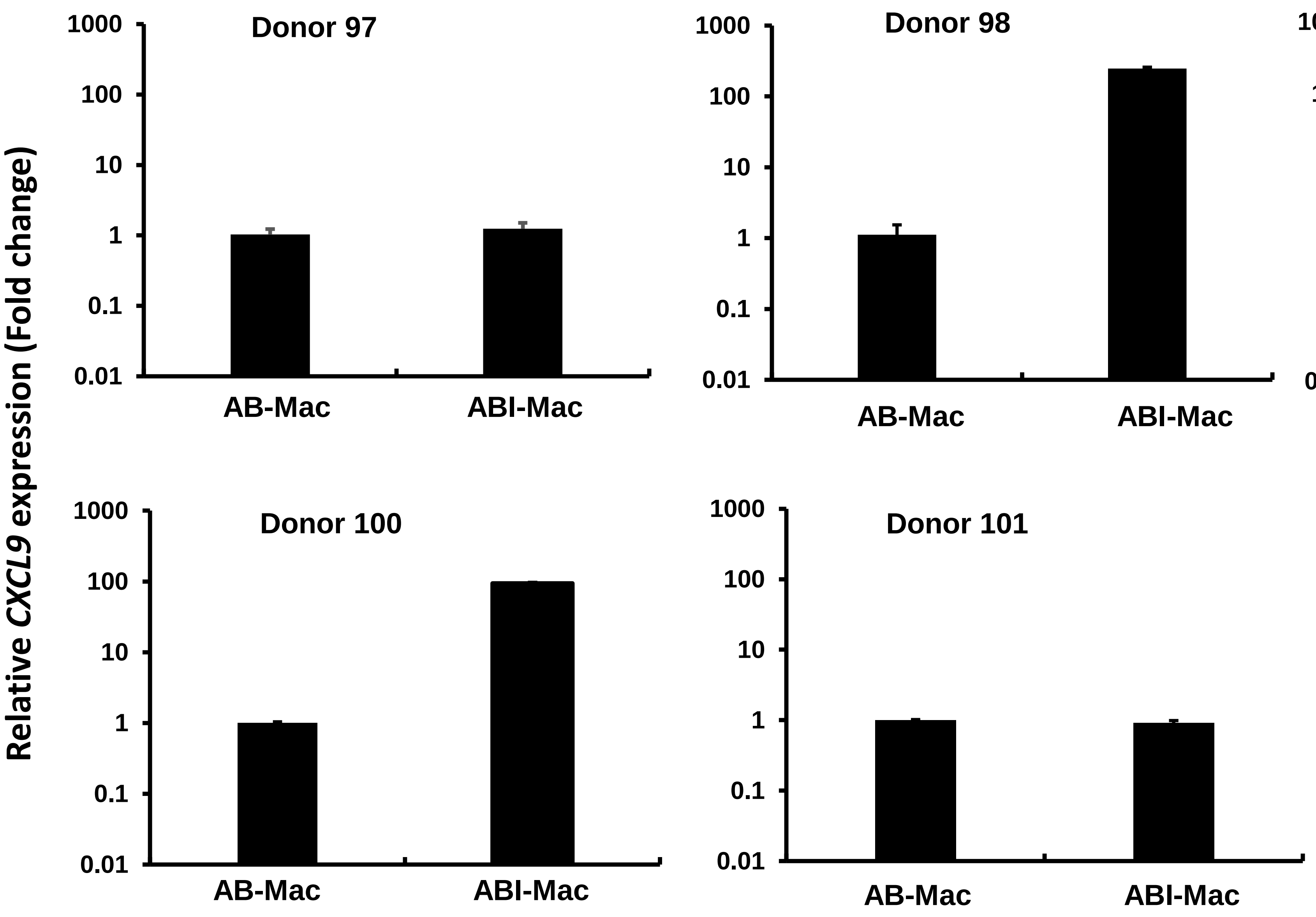

S. Figure S2: Evaluation of the expression of CXCL9 gene.
Total cellular RNA was extracted from AB- and ABI-Mac of five independent donors (Donor 97 ~ 101), and then real time RT-PCR was performed using CXCL9 probe. *CXCL9* expression in ABI-Mac was compared with that in AB-Mac and the gene expression is indicate fold change. Data represents a result from two independent assay. Data shown represent mean ± SD.

S. Figure S3

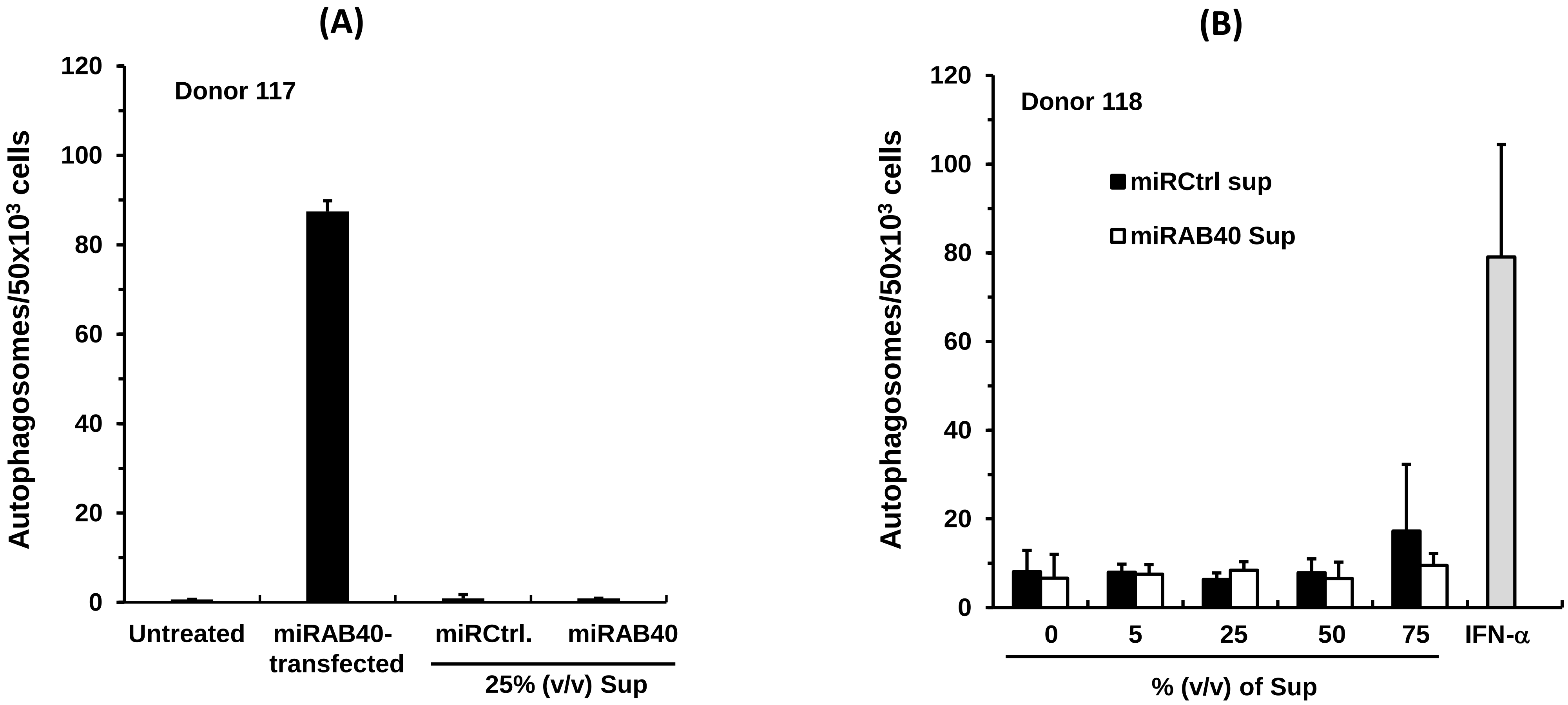

S.Fig. S3. Impact of culture supernatants on autophagy induction. Fresh M-Macs from two independent donors (Donor 117 and 118) were cultured with 25 (v/v) % (A) or with 0 - 75% (v/v) (B) of cell-free culture supernatants derived from miRCtrl- or miRAB40-transfected cells (Donor 111) three days. Autophagosome induction was quantified as described in the Materials and Methods. As a positive control, miRAB40-transfected (A) and IFN (100 units/ml) treated M-Macs were included in the assays. Data presents mean ± SD (*n*=3)

S. Figure S4

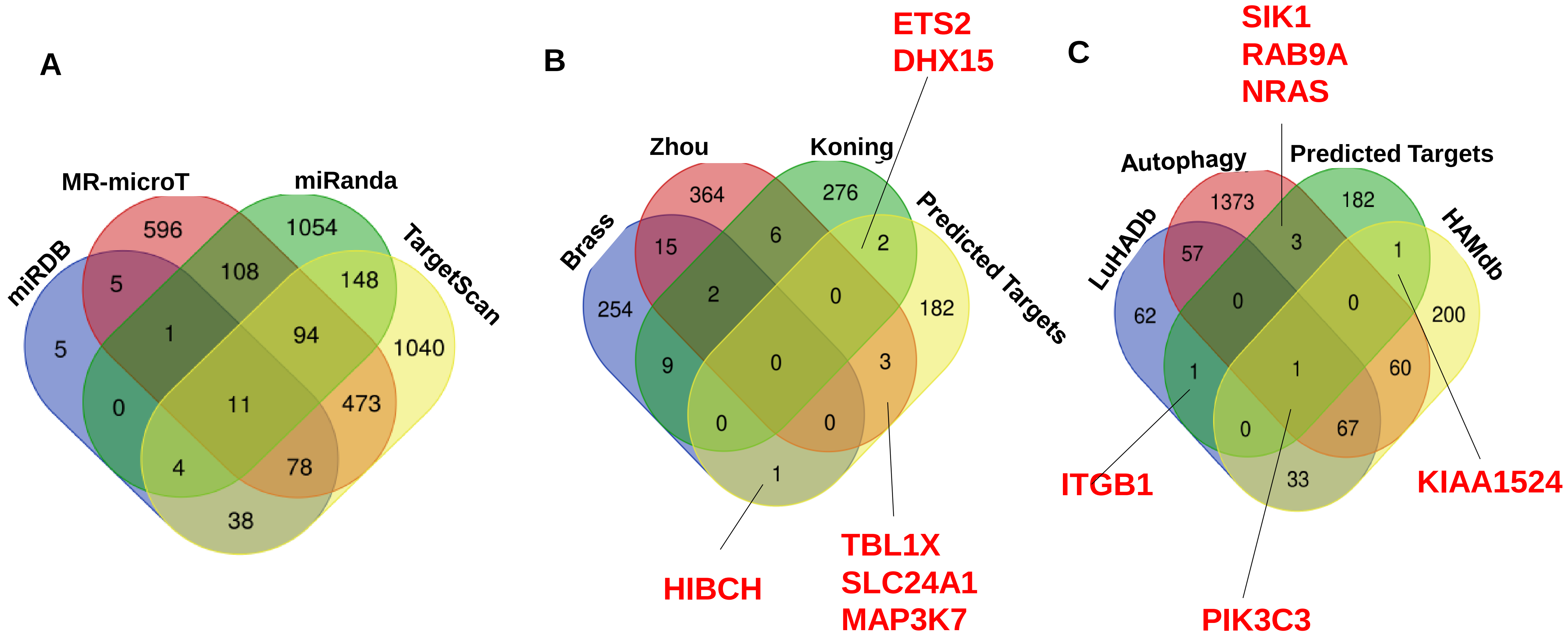

S. Figure S4. Target prediction analysis of miRAB40. (A) Target prediction analysis for miRAB40 were performed using miRDB, MR-microT, miRanda, and Target Scan as described in the Materials and Methods. To identify commonly predicted target genes, Venn diagram analysis was performed. (B, C) The predicted genes of miRAB40 were compared with HDFs (B) or autophagy data bases (C). The genes overlapped with the predicted target genes and each data base are listed in the diagrams.

S. Figure S5

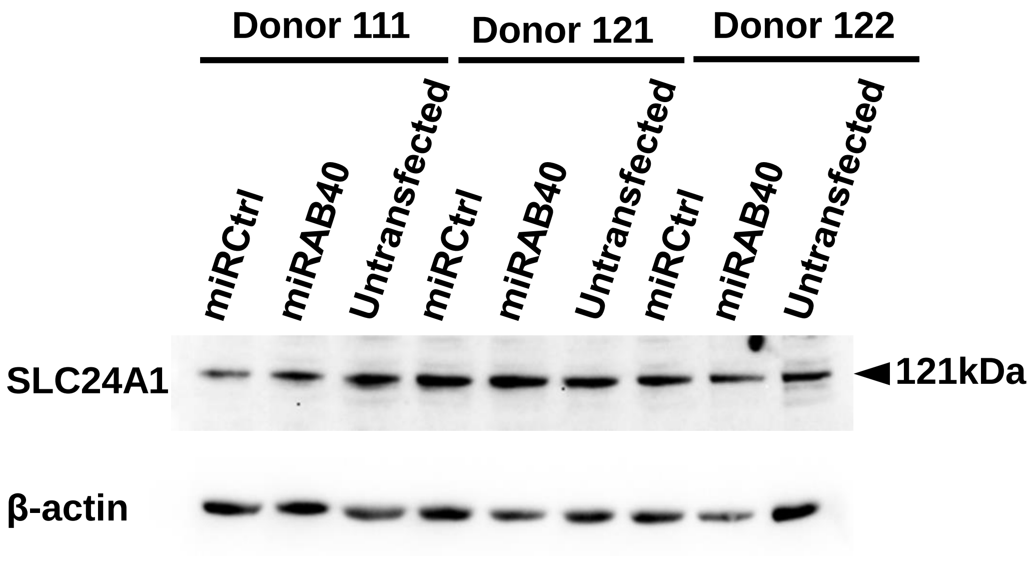

S. Figure 5. Western blot analysis of SLC24A1 expression. M-Macs from three different donors (Donor 111, 121, 122) were transfected with miRCtrl or miRAB40 and cultured for three days. Cell lysates were made with radioimmune precipitation assay buffer (Boston Biology), and protein amounts were quantified using the BCA protein assay kit (Thermo Fisher). A total of 20 µg of cellular protein was separated on a 4–12% Bis-Tris gel (Thermo Fisher) in MOPS running buffer (Thermo Fisher) and Western blot was performed with anti-SLCA24A1 antibody (Cat# NBP2-20384, Novus biologicals, Littleton, CO, USA). As an internal loading control, anti-b actin (Sigma Aldrich) was used. Protein bands were detected with ECL plus Western blotting detection reagents (GE Healthcare).

S. Figure S5 original

miRAB40 anti-HIV related

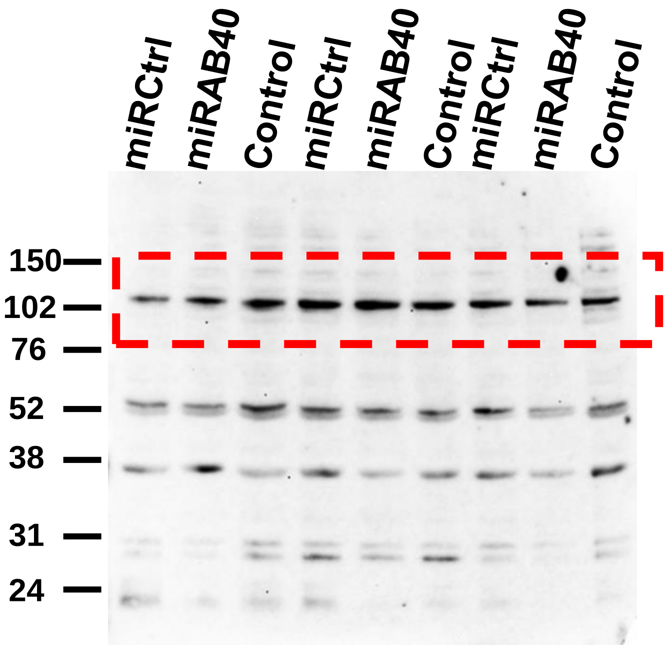

These bands were used in the S.Fig5

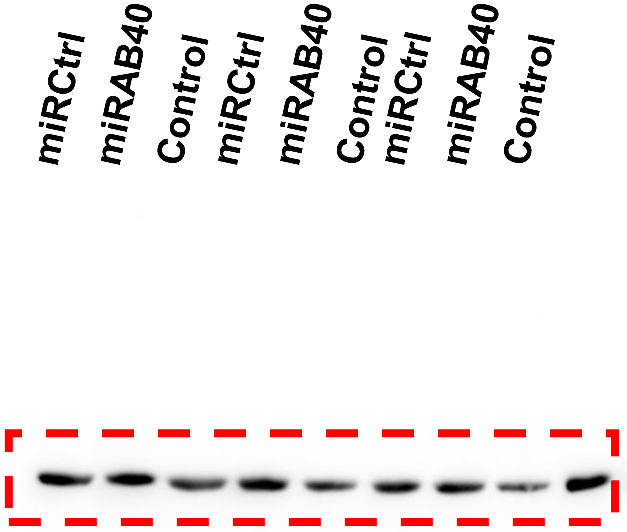

Supplement: Supplementary file 1 [file ijms-22-01290-s001.zip › Supplementary files/Rev.S.Fiugre S1_to_S5_Sura.pdf]
